# Analysis of genetic variants *TREM2* and plasma gingipain K and R level in patients with Alzheimer’s disease

**DOI:** 10.1002/alz.091883

**Published:** 2025-01-03

**Authors:** Mikolaj Hurla, Natalia Banaszek, Jolanta Florczak‐Wyspianska, Olivia Michalczyszyn, Wojciech Owecki, Wojciech Kozubski, Jolanta Dorszewska

**Affiliations:** ^1^ Laboratory of Neurobiology, Department of Neurology, Poznan Poland; ^2^ Poznan University of Medical Sciences, Poznan Poland; ^3^ Chair and Department of Neurology, Poznan Poland

## Abstract

**Background:**

Alzheimer’s disease (AD) is characterized by an acquired, progressive impairment of cognitive functions. The pathogenesis of this disease remains unknown. It is explained based on the following theories: amyloid cascade, inflammation, vascular, and infection hypothesis. Recently hypothesized that Porphyromonas gingivalis may be a unifying factor linking the different hypotheses related to AD pathogenesis. P. gingivalis produces cysteine proteinases called gingipain K and gingipain R, which are virulence factors. P. gingivalis plays a significant role in periodontitis. Periodontitis is provoked by bacterial lipopolysaccharide, which then leads to an immune response. The *TREM2* gene is involved in the immune response. P. gingivalis may be responsible for reducing the expression of the *TREM2* gene and TREM2 protein. TREM2 protein deficiency may impair Aß degradation, which may significantly increase the risk of AD. It has been showed a tendency for earlier onset of symptoms and faster progression of AD in people who were carriers of the potentially pathogenic *TREM2* (rs143332484) CT variant of this gene. The study aimed to analyze plasma gingipain K and R concentration and *TREM2* (rs143332484) genetic variants in AD patients, control subjects related to AD cases (CR), and control subjects without a family history of AD (CU).

**Method:**

The studies were conducted on 74 subjects (AD and controls). The *TREM2* genotype was determined by HRM and sequencing. The gingipain K and R concentrations were determined by the ELISA method.

**Results:**

The highest concentration of gingipain K (p = 0.0820) and R (p = 0.0740) was found in AD compared to CR, and CU. The *TREM2* CT genotype was most common in AD patients. Patients with this genotype had the highest concentration of gingipain K. However, AD patients with the *TREM*2 CC genotype showed statistically significantly higher concentrations of gingipain R (p<0.05) compared to CR and CU.

**Conclusion:**

It seems that both gingipains K and R may be involved in the pathogenesis of AD.

